# Study on Design and Preparation of Conductive Polyvinylidene Fluoride Fibrous Membrane with High Conductivity via Electrostatic Spinning

**DOI:** 10.3390/polym15153174

**Published:** 2023-07-26

**Authors:** Xinhua Zhao, Qian Zhao, Yanjiao Chang, Mingzhuo Guo, Siyang Wu, Hanqi Wang, Yihao Hou, Luyu Zhang, Chang Liu, Han Wu, Yunhong Liang, Luquan Ren

**Affiliations:** 1 The Key Laboratory of Bionic Engineering, Ministry of Education, Jilin University, Changchun 130025, China; 2 College of Food Science and Engineering, Jilin University, Changchun 130062, China

**Keywords:** electrostatic spinning, conductive fibrous membrane, polyvinylidene fluoride, porous structure, silver particles

## Abstract

The novel conductive polyvinylidene fluoride (PVDF) fibrous membrane with high conductivity and sensitivity was successfully prepared via electrostatic spinning and efficient silver reduction technology. Based on the selective dissolution of porogen of polyvinylpyrrolidone (PVP), the porous PVDF fibrous membrane with excellent adsorbability and mechanical strength was obtained, providing a structure base for the preparation of conductive PVDF fibrous membrane with silver nanoparticles (AgNPs-PVDF). The Ag^+^ in the AgNO_3_ mixed solution with PVP was absorbed and maintained in the inner parts and surface of the porous structure. After the reducing action of ascorbic acid-mixed solution with PVP, silver nanoparticles were obtained tightly in an original porous PVDF fibrous membrane, realizing the maximum conductivity of 2500 S/m. With combined excellent conductivity and mechanical strength, the AgNPs-PVDF fibrous membrane effectively and sensitively detected strain signals of throat vocalization, elbow, wrist, finger, and knee (gauge factor of 23). The electrospun conductive AgNPs-PVDF combined the characteristics of low resistance, high mechanical strength, and soft breathability, which provided a new and effective preparation method of conductive fibers for practical application in wearable devices.

## 1. Introduction

In recent years, electronic skin and wearable electronic devices have shown great potential application in various fields such as human health monitoring and human–machine interaction [1,2,3,4,5,6,7,8,9,10]. These technologies offer excellent sensing capabilities, which can be seamlessly integrated into daily life. However, there are still challenges to overcome in terms of materials and performance to ensure their successful implementation. Polymer-based flexible and lightweight electronic skins, particularly those using hydrogels, have received extensive research attention. These materials exhibit desirable properties such as flexibility, elasticity, and lightweight [11,12,13,14,15,16,17]. However, factors of sweat evaporation, oxygen permeability, and heat diffusion are often overlooked. Portable sensors designed for human motion detection should ensure performance requirements of sufficient flexibility and breathability. Recognizing the limitations of current materials, researchers have turned their attention to fabric-based sensors. Fabric-based sensors provide wearable advantages and a more natural interface with the human skin [18,19,20,21,22,23]. However, traditional textile materials are typically insulating and hard to be directly conductive in smart textiles. Therefore, finding effective methods to integrate conductive materials with fabrics is crucial for the realization of fabric-based sensing applications.

In the early stages of research, silver, nickel, aluminum, and copper products were primarily used as conductive fillers. To achieve better performance, conductive inks, carbon-based conductive polymers, intrinsic conductive polymers, and conductive polymers were employed in smart fabrics. Among the various methods, electrodeless plating and specific adsorption have emerged as promising and cost-effective approaches, which can enable the uniform distribution of silver nanoparticles on the surface of fibers. Moreover, the method can also ensure strong adhesion and minimize the risk of detachment of nanoparticles [24,25]. Corresponding research reported the successful preparation of composite fibers with uniformly loaded silver nanoparticles through electrochemical plating and specific adsorption methods.

Among the methods for preparing functional fibers, electrostatic spinning possesses the advantages of simple equipment, convenient operation, wide application scales, and high efficiency, which has prepared many kinds of fibers with high specific surface area, permeability, and absorption efficiency. Moreover, the characteristics of electrospun fibers can also be improved via the control of the electrospun process. Therefore, electrostatic spinning is widely used in the fields of biomedicine, filtration materials, composite materials, catalysts, food science, and so on. Another important part of conductive electrospun fibers is the selection and addition method of conductive materials. The silver matrix conductive materials own steady physical and chemical properties, which are the common conductive materials in strain sensors. As the main matrix of functional fibers, the electrospun fibers with relatively high mechanical strength, elasticity, and durability are the functional base of conductive fibers. In the material candidates of electrospun solutions, polyvinylidene fluoride owns excellent thermal stability, chemical stability, and mechanical properties, which can realize various different fiber structures and dimensions. The electrospun polyvinylidene fluoride fibers provide an effective candidate for the preparation of conductive fibers. Considering a combination conductive material of silver and electrospun fibers, the methods which can realize the conductive property of fibers are the spraying method, vacuum filtration method, ultrasonic induction method, in situ polymerization method, electroless plating method, and so on. Even though the above methods can realize the electric conduction of fibers, the combination of high conductivity of electrospun fibers, high mechanical strength, and high electric sensing sensitivity need to be improved further.

This study aims to solve the limitations of conductive electrospun fibers with high conductivity and sensitivity, which improves the advancement of wearable electronic devices and fabric-based sensing applications. In order to achieve this goal, composite fibers were prepared using electrospinning technology. By utilizing the stability of polyvinylidene fluoride (PVDF) and the water solubility of PVDF-PVP, porous fibers were successfully prepared. These fibers were then subjected to the adsorption of silver nitrate ions, followed by reduction, resulting in fibers with silver ions attached to both their surfaces and pores. Subsequently, the fibers were immersed in a reducing solution, leading to the generation of fibers with silver particles attached to surfaces. Moreover, the conductivity of the electrospun fibers was controlled by adjusting the concentration of the silver nitrate solution. Additionally, the strain sensors were used to monitor the movements of various joints, verifying the feasibility and efficiency of the preparation method of conductive polyvinylidene fluoride fibrous membrane with high conductivity via electrostatic spinning.

## 2. Experimental Section

### 2.1. Materials

Polyvinylidene fluoride (PVDF, Kynar 761) was purchased from France Arkema Co., Ltd. (Paris, France). Dimethyl Formamide (DMF), acetone and silver nitrate (AgNO_3_) were purchased from Tianjin Beilian Fine Chemicals Development Co., Ltd. (Tianjin, China). Polyvinylpyrrolidone K30 (PVP) and ascorbic acid (VC, C_6_H_8_O_6_) of analytical grade were purchased from Xi’an Yuelai Pharmaceutical Excipients Co., Ltd. (Xi’an, China) and Tianjin Guangfu Technology Development Co., Ltd. (Tianjin, China), respectively. All materials were used without further purification. Deionized water was used as the solvent for all experiments.

### 2.2. Preparation of Conductive Fibrous Membrane

#### 2.2.1. Preparation of Porous PVDF Fibrous Membrane

To prepare a uniform precursor solution for electrospinning, DMF and acetone with a volume ratio of 6:4 were added into a three-necked flask. Then, 1.554 g PVDF was also added into the mixed solution. In order to investigate the effect of PVP content on absorption and mechanical strength of porous PVDF fibrous membrane, the weight percentages of PVP in the solution were 1 wt.%, 5 wt.%, 10 wt.%, 15 wt.%, and 20 wt.%. The mixture was thoroughly mechanically stirred at 500 r/min for 2 h in a 50 °C oil bath environment. The resulting precursor solution was transferred to two injectors and fixed on both sides of the electrospinning machine for conjugate spinning. The rotation speed of the drum collector was 80 rpm. The electrospinning positive and negative voltages were +8 kV and −8 kV, respectively. The spinning distance and height were set as 12 cm and 6 cm, respectively. After spinning, the obtained fibrous membrane was cut into appropriate sizes and immersed in deionized water for 3 h to remove the PVP, obtaining the porous PVDF fibrous membrane.

#### 2.2.2. Preparation of AgNPs-PVDF Conductive Fibrous Membrane

Firstly, 100 mL solution of PVP/deionized water with a mass ratio of 1:9 was prepared as a stabilizer. Silver nitrate was dissolved in deionized water to form AgNO_3_ solutions with concentrations of 0.6, 0.8, 1.0, 1.2, and 1.4 mol/L. The stabilizer was mixed in equal amounts with the AgNO_3_ solution to form an AgNO_3_/PVP mixed solution. Water on the porous PVDF fibrous membrane was removed by filter paper. Then, the membrane was soaked in the AgNO_3_/PVP solution for 3 h to fully absorb the silver ions.

**Figure 1:**
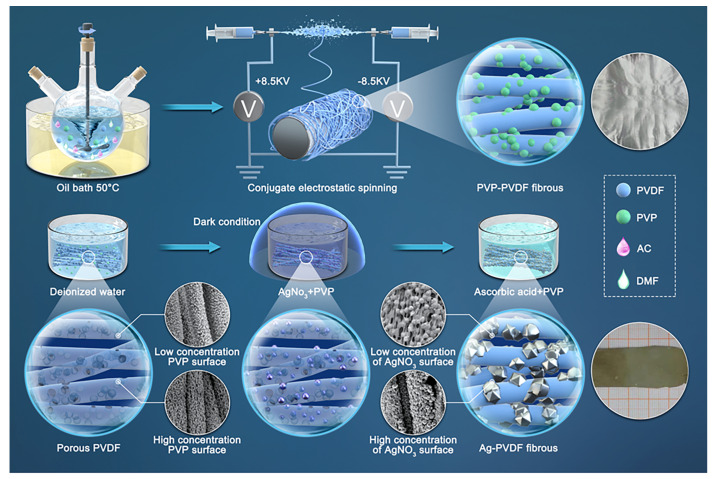
Preparation process diagram of conductive porous PVDF and AgNPs-PVDF fibrous membrane.

A total of 1.41 g ascorbic acid was dissolved in 100 mL deionized water and mixed with equal amounts of PVP to form a VC/PVP mixed reduction solution. The porous PVDF fibrous membrane with silver ions was soaked in the reduction solution for 3 h. The AgNO_3_ was reduced to Ag nano particles (AgNPs), forming the AgNPs-PVDF conductive fibrous membrane. The whole preparation process of AgNPs-PVDF is shown in Figure 1.

### 2.3. Characterization and Measurements

#### 2.3.1. Microstructure and Phase Component Analysis

The porous PVDF and AgNPs-PVDF fibrous membrane were observed by field-emission scanning electron microscopy with energy dispersive spectroscopy to observe the microstructure and distribution pattern of reduced silver nanoparticles. The phase components of the AgNPs-PVDF fibrous membrane were obtained by X-ray diffraction (XRD) with Cu Kα radiation in the range of 10–90°, and the scan speed was set to 4 deg/min.

#### 2.3.2. Fourier Transform Infrared Spectrum (FTIR) Analysis

FTIR is commonly employed for investigating functional groups of fibrous membranes. Specifically, the attenuated total reflection (ATR) mode of an infrared spectrometer (Nicolet i0, Waltham, MA, USA) is utilized to obtain vibrational spectra of samples within the range of 500–4000 cm^−1^ wavelengths with the resolution of 4 cm^−1^ and 32 scans per sample. 

#### 2.3.3. Mechanical Strength Analysis

The mechanical strength of the PVDF matrix fibrous membranes was tested via a universal testing machine with a loading rate of 50 mm/min at room temperature. The samples were resized to 50 mm in length, 20 mm in width, and 0.5 mm in height. Each group was tested using at least three samples.

The cyclic tensile tests were conducted at room temperature to evaluate the durability of PVDF matrix fibrous membranes. Sample size was 50 mm in length, 20 mm in width, and 0.5 mm in height. The clamp distance was 30 mm. The sensing characteristics were obtained under the conditions of specific loading rate or strain values. The loading rates were set as 20, 50, 100, 200, 400, 600, 800, and 1000 mm/min. The strain values were set as 10, 15, 20 and 25%.

#### 2.3.4. Differential Scanning Calorimetry (DSC) Analysis

DSC was utilized to determine the reduction amount of silver in AgNPs-PVDF conductive fibrous membranes with various concentrations of silver nitrate. The testing temperature range was 20–800 °C. The heating rate was 10 °C/min. The entire heating and cooling process was safeguarded by a nitrogen flow.

#### 2.3.5. Porosity Analysis

The porosity of porous PVDF fibrous membrane was the volume percentage of the pores in a unit volume of fibrous membrane. The porosity was calculated by the following the equation:

(1) $$Ρ=\left(1-\frac{m}{\rho sh}\right)\times 100\%$$

where *P* was the fibrous membrane porosity; *m* (g) was the gram weight of the fibrous membrane; *S* (cm^2^) was the area of the fibrous membrane; *h* (cm) was the thickness of the fibrous membrane; *ρ* (g/cm^−3^) was the density of the PVDF powder.

#### 2.3.6. Electrical Conductivity Test

The electrical conductivity and gauge factor of the AgNPs-PVDF fibrous membrane were evaluated. The samples were cut into neat rectangles with a dimension of 50 mm × 20 mm × 0.5 mm. The voltage (U) and current (I) applied to the samples were monitored in real-time via a digital display DC power supply. The resistance of the samples was determined via Ohm’s law (*R* = U/I). The electrical conductivity of the fibrous membrane (*σ*, S m^−1^) was calculated by the following equation:

(2) $$\sigma =\frac{L}{RA}$$

where *L* (m) is the test distance; *R* (Ω) is the resistance of the fibrous membrane; *A* (m^2^) is the cross-sectional area of the fibrous membrane.

For the gauge factor test, the fibrous membrane was fixed on a universal testing machine with a stretching rate of 30 mm/min. The resistance values were recorded by a digital display multimeter (KEYSIGHT, 34465A, Santa Rosa, CA, USA) with an NPLC of 0.02. The size of the tested sample was similar to the tensile sample. The relative change in the resistance was calculated by the following equation:

(3) $$\Delta R=R-R_{0}$$

where *R* and *R*_0_ represented the real-time and initial resistance, respectively.

The gauge factor (*GF*) was calculated by the following equation:

(4) $$GF=\frac{\Delta R}{\varepsilon R_{0}}$$

where *R*_0_ and Δ*R* represent the original resistance without deformation and the resistance variation with a specific strain *ε*, respectively.

#### 2.3.7. Electrical Sensing Function Test

AgNPs-PVDF conductive fibrous membrane was connected to an LED circuit of “JLU” (Jilin University) style, powered by an MS305D adjustable DC-regulated power supply. The voltage was 2.0 V. The AgNPs-PVDF conductive fibrous membranes were attached to various joints of fingers, vocal cords, wrists, elbows, and knees. The electric signal generated from joint motion was detected and recorded in real-time via a Keysight-36645A digital precision multimeter.

## 3. Results and Discussion

### 3.1. Characteristics of Porous PVDF Fibrous Membrane

**Figure 2:**
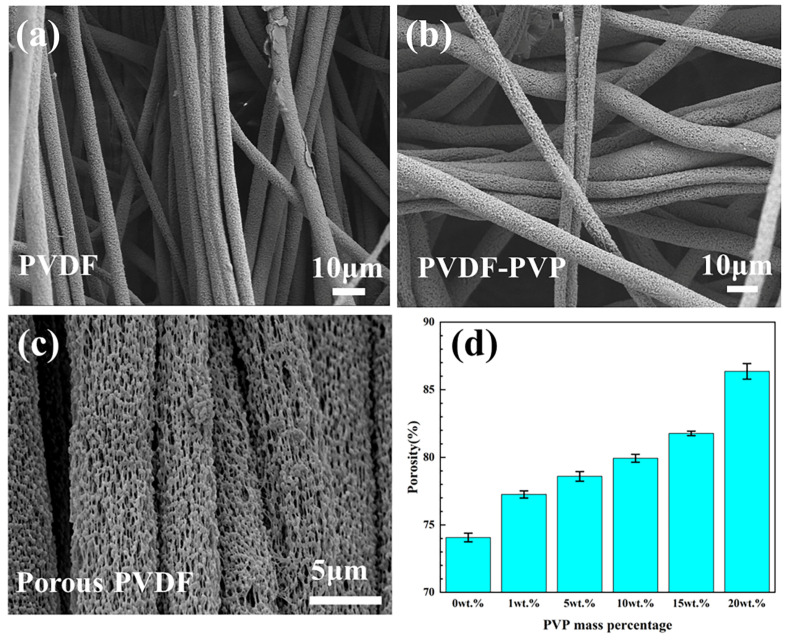
Microstructure of (**a**) PVDF, (**b**) PVDF-PVP, and (**c**) porous PVDF fibrous membranes and (**d**) porosity of porous PVDF with various PVP contents.

To evaluate the effect of PVP content on the performance of porous PVDF fibrous membranes, various analyses including microstructure morphology, porosity, mechanical strength, and FTIR tests were conducted. Figure 2 illustrates the intuitionistic effect of PVP content on the microstructure of porous PVDF. Figure 2a exhibits the scanning electron microscopy (SEM) microstructure of PVDF fibrous membranes. The diameter of the monoradicular PVDF fiber with orientation was about 4 μm. Figure 2b shows the microstructure of PVDF with PVP. The addition of PVP maintained the original diameter of monoradicular PVDF fiber. But, the surface of the PVDF-PVP fibrous membrane was coarser than that of PVDF. The removal of PVP from the PVDF fibrous membrane led to the uniform porous structure on the surface. Figure 2c demonstrated that the porosity of the porous PVDF fibrous membrane increased gradually with the increase in PVP mass fraction. The porosity of the membrane is 73.9% when the PVP content is 0 wt.%. However, with the increase in PVP content from 1 wt.%, 5 wt.%, 10 wt.%, 15 wt.% to 20 wt.%, the porosity increased to 77.1%, 78.2%, 79.4%, 81.2%, and 86.3%, respectively, as shown in Figure 2d. Notably, the significant increase in porosity occurred in the range from 15 wt.% to 20 wt.%. Combined with the SEM microstructure analysis, PVP content controlled the porosity of the porous PVDF fibrous membranes.

**Figure 3:**
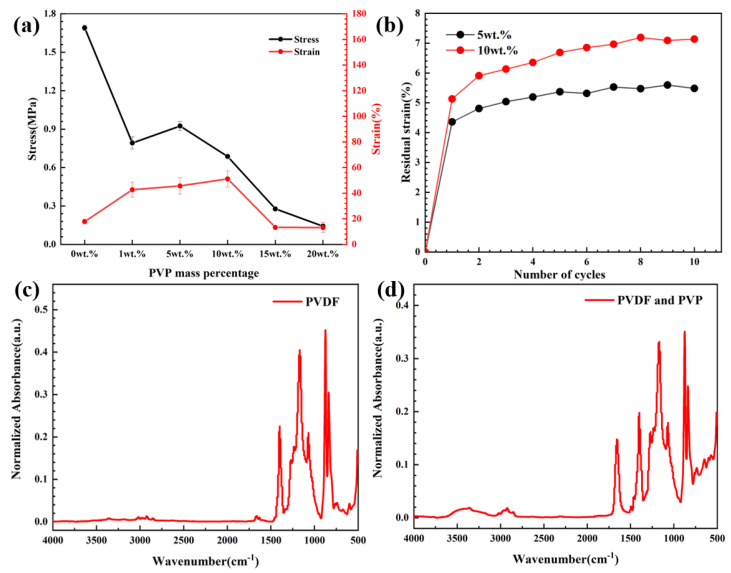
The (**a**) stress and strain values, (**b**) residual strain of porous PVDF with various PVP content and FTIR of (**c**) PVDF and (**d**) PVDF-PVP fibrous membrane.

Figure 3a displays the stress and strain values of the porous PVDF fibrous membrane under various porosity conditions. The maximum stress and strain values of the PVDF fibrous membrane were 1.69 MPa and 17.83%, respectively. With the increase in PVP contents, the stress of the porous PVDF fibrous membrane increased first and then decreased. When the PVP content was 5 wt.%, the porous PVDF reached the peak value of 0.93 MPa and then decreased to the minimum value of 0.14 MPa at 20 wt.%. The strain initially shows a gradual increase, which reached the maximum value of 13.28% at 15 wt.% and then decreased to the minimum value of 13.19% at 20 wt.%. Based on the stress–strain curve of the porous PVDF fibrous membrane, although the PVDF fibrous membrane endured the highest stress without PVP, it exhibited a lower strain. With the increase in PVP content, the porous PVDF fibrous membrane demonstrated a better strain value within the range from 1 wt.% to 10 wt.%. When the PVP content exceeded to 15 wt.%, the porous PVDF fibrous membrane was prone to fracture. The porous structure on the fiber surface was discontinuous micropores with a low PVP content. The micropores were interconnected on the base of high PVP content, leading to strength reduction. Figure 3b shows the residual strain of the porous PVDF which owned excellent stress and strain performance. The experiment manifested that the porous PVDF fibrous membrane with a PVP content of 5 wt.% owned the highest mechanical strength and elasticity.

Figure 3c,d display the FTIR analysis of PVDF fibrous membrane and porous PVDF fibrous membrane with PVP. The results showed that the addition of PVP introduced new functional groups in the porous PVDF fibrous membrane, which can be found by the emergence of a characteristic peak of -C-N- at 1272.72 cm^−1^ and a characteristic peak of -C=O within the range of 1660–1680 cm^−1^. Compared with the -C-H stretching vibration peaks at 2900–3000 cm^−1^, the higher PVP absorption peaks at 3000 and 3500 cm^−1^ confirmed that the hydrophilic characteristics of the PVDF fibrous membrane were altered by the addition of PVP, which built an absorption base of silver ions. The addition of PVP maintained the original functional groups of PVDF and provided function base of porous structure for the preparation of conductive PVDF fibrous membrane. Combined with mechanical strength, the PVP content of 5 wt.% was selected as the optimal content of the preparation of porous PVDF fibrous membrane. 

### 3.2. Characteristics of Conductive Porous PVDF

Multiple experiments including microscopic morphology, differential thermal analysis, conductivity, and FTIR analysis were carried out to investigate the effect of concentration variation in silver nitrate on the properties of AgNPs-PVDF conductive fibrous membranes and disclosed the optimal preparation parameters of the reduction reaction of silver particles.

**Figure 4:**
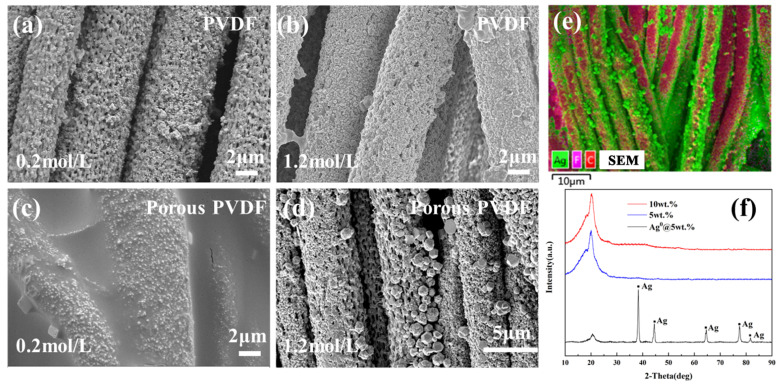
Microstructure of conductive PVDF fibrous membrane with (**a**) 0.2 mol/L and (**b**) 1.2 mol/L and conductive porous PVDF fibrous membrane with (**c**) 0.2 mol/L and (**d**) 1.2 mol/L, (**e**) energy spectrum and (**f**) XRD of AgNPs-PVDF conductive fibrous membrane.

Figure 4a,b show the microstructure of PVDF fibrous membranes with various concentrations of silver nitrate solution after reduction reaction. Silver particles of small size and amount are distributed sparsely on surfaces of PVDF fibrous membrane. When the concentration of silver nitrate solution increased to 1.2 mol/L, the amount of silver particles with a relatively small size was increased. Figure 4c,d show the microstructure of porous PVDF fibrous membranes with various concentrations of silver nitrate solution after the reduction reaction. A large amount of silver particles with a small size were distributed uniformly on surfaces of the AgNPs-PVDF fibrous membrane. When the concentration of silver nitrate solution increased to 1.2 mol/L, a large amount of silver particles of a big size existed on surfaces of the AgNPs-PVDF fibrous membrane. The higher the concentration of the silver nitrate solution was, the denser and higher the amount of silver particles of a big size existed. Combined with the energy spectrum in Figure 4e and XRD curves in Figure 4f, the particles on PVDF surfaces and AgNPs-PVDF surfaces were a silver elemental substance.

Based on the microstructure and phase component analysis of silver particles on the AgNPs-PVDF fibrous membrane, the method of reduction reaction of silver particles was effective. Compared with PVDF fibrous membrane, the porous structure owned a large amount of silver particles with a bigger size, which proved the effectiveness of the preparation of porous structures. The large amount of dense silver elemental substance bonded tightly with the porous PVDF fibrous membrane and provided high conductivity and durability for the application of the AgNPs-PVDF fibrous membrane.

**Figure 5:**
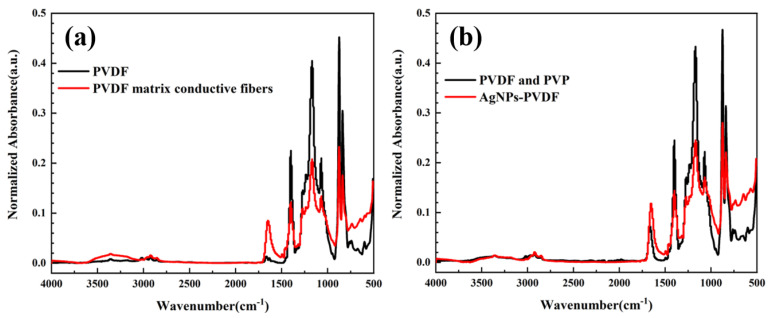
FTIR of (**a**) PVDF and conductive PVDF fibrous membrane and (**b**) PVDF with PVP and AgNPs-PVDF fibrous membrane.

In order to verify whether the reduction reaction of silver particles affected the functional groups of the AgNPs-PVDF fibrous membrane or not, FTIR analysis of PVDF and conductive PVDF fibrous membrane, and PVDF with PVP and the AgNPs-PVDF fibrous membrane were conducted, as shown in Figure 5. The PVDF and porous PVDF fibrous membranes owned -C=O characteristic peaks in the range of 1660–1680 cm^−1^ and -C-N- characteristic peaks at 1272 cm^−1^. After the reduction reaction, -C=O and -C-N- characteristic peaks were also existed in FTIR curves. Based on FTIR results, it can be found that reduction reaction of silver particles only realized conductivity of the AgNPs-PVDF fibrous membrane. There were no new functional groups existed in the AgNPs-PVDF fibrous membrane. The reduction reaction of silver particles maintained the original material characteristics of porous PVDF and realized conductive base.

**Figure 6:**
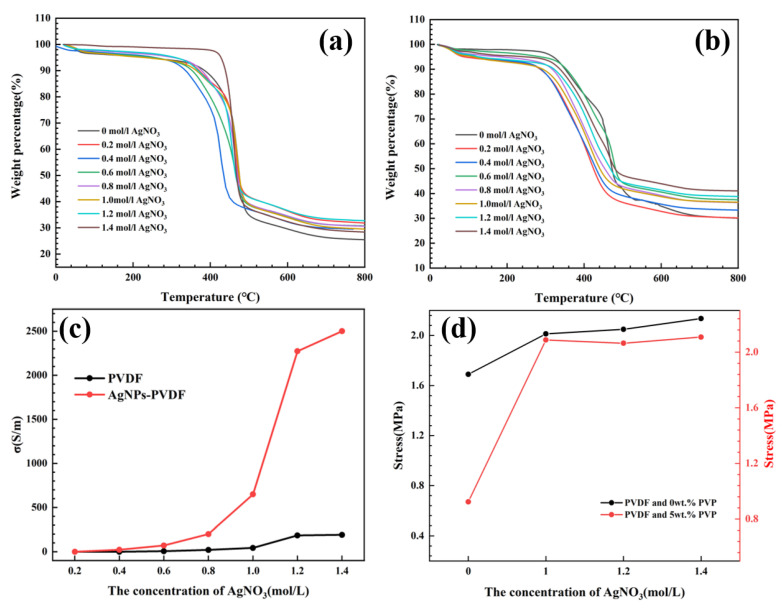
Differential thermal analysis of (**a**) PVDF and (**b**) AgNPs-PVDF conductive fibrous membranes with different AgNO_3_ concentrations, and (**c**) conductivity and (**d**) mechanical strength of PVDF and AgNPs-PVDF conductive fibrous membranes.

Effects of silver nitrate concentration on the adsorption capacity and stability of reduced silver nanoparticles was investigated by the differential thermal analysis of PVDF and AgNPs-PVDF conductive fibrous membranes. Compared with Figure 6a,b, the residual amount of AgNPs-PVDF (30.04%) after 800 °C heat treatment was higher than that of PVDF (25.44%) conductive fibrous membranes. Therefore, the porous structure of porous PVDF effectively absorbed higher amount of silver ions, which proved the feasibility of the porous structure. Figure 6c displays the conductivity of PVDF and AgNPs-PVDF conductive fibrous membranes after reduced reactions with different concentrations of silver nitrate, which exhibited the positive correlation between the concentration of silver nitrate and the conductivity of the fibrous membrane. In the concentrations of 0.2–0.4 mol/L, the conductivity of the PVDF and AgNPs-PVDF fibrous membrane were approximately zero, exhibiting a high resistance and insulating behavior. In the range of 0.8–1.2 mol/L, the conductivity of the AgNPs-PVDF fibrous membrane increased rapidly, reaching 2272 S/m at 1.2 mol/L. In the range of 1.2–1.4 mol/L, the conductivity of the AgNPs-PVDF fibrous membrane increased gradually and attained a maximum conductivity of 2500 S/m. The conductive PVDF fibrous membrane still maintained a low conductivity in the range of 0–1.4 mol/L. Considering the effects of Moore’s law and the marginal effect on experiments, 1.2 mol/L was selected as the optimal concentration of silver nitrate for the reduction reaction of silver particles. Based on the conductivity comparation analysis of the PVDF and AgNPs-PVDF fibrous membrane, the method of conductive PVDF fibrous membrane was accurate, which maintained the original material characteristics of PVDF and combined high conductivity.

Combined with Figure 2, Figure 3, Figure 4, Figure 5 and Figure 6, the construction of the porous structure with excellent absorption and mechanical strength and the preparation method of conductive AgNPs-PVDF fibrous membranes with excellent conductivity, mechanical strength, and bonding strength between silver particles and porous PVDF matrix are effective and feasible. Based on excellent conductivity and mechanical properties, the AgNPs-PVDF fibrous membrane owns the advantages of low resistance and strain-sensing properties. Therefore, the practical conductivity and sensing properties of the AgNPs-PVDF fibrous membrane were investigated to extend the application fields of electrospun conductive fibrous membrane.

### 3.3. Characteristics of Strain Sensing Property

**Figure 7:**
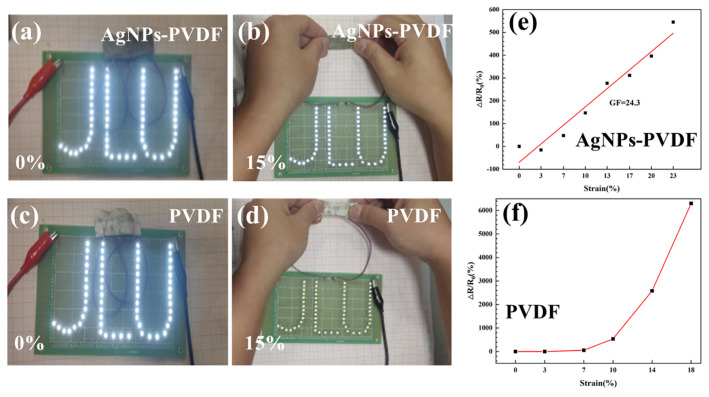
Conductive effect of (**a**) AgNPs-PVDF conductive fibrous membrane without strain and (**b**) with 15% strain, and (**c**) PVDF conductive fibrous membrane without and (**d**) with 15% strain, and (**e**) ΔR/R_0_ variation in AgNPs-PVDF conductive fibrous membrane and (**f**) PVDF conductive fibrous membrane.

The conductive AgNPs-PVDF fibrous membrane was connected to the LED circuit, which operated at a rated voltage of 2.0 V. As depicted in Figure 7a, the LED light emitted a satisfactory level of illumination. When the AgNPs-PVDF fibrous membrane was subjected to a 0.5 cm displacement (15% strain), the brightness of the LED decreased, as shown in Figure 7b. Compared with Figure 7a,b, the PVDF conductive fibrous membrane can also light the LED circuit with a rated voltage of 2.0 V in Figure 7c. But, the LED circuit was extinguished with tensile strain of 15%, as shown in Figure 7d. Therefore, besides excellent conductivity, the prepared AgNPs-PVDF fibrous membrane owned excellent strain conductivity, which was not the characteristic of the PVDF conductive fibrous membrane.

Δ*R*/*R*_0_ curves of the strain sensitivity of the AgNPs-PVDF conductive fibrous membrane are presented in Figure 7e. Prior to reaching the critical strain of 25%, the strain sensitivity of the conductive fibrous membrane obtained via 1.2 mol/L AgNO_3_ solution was 24.3. Based on the fitted curve of ΔR/R_0_ variation, the prepared AgNPs-PVDF conductive fibrous membrane owned excellent linearity and sensitivity. Before the strain of 20%, the Δ*R*/*R*_0_ variation in PVDF conductive fibrous membrane exhibited low sensitivity and linearity in Figure 7f, which cannot reflect the real strain variation accurately. The sensing ability indicated that the disconnection and reconstruction of the conductive network was a dynamic process under strain load, manifesting as resistance fluctuations in the macroscopic realm. Consequently, the variation in the Δ*R*/*R*_0_ values can be considered the primary indicator in the strain-sensing test process. During the stretching of the conductive fibrous membrane, changes occurred in its length and width. As the stretching was performed along the axial direction of the conductive fibers, the length of the fibrous membrane increased while the width simultaneously decreased. In the strain range of 0–3%, the membrane width rapidly decreased due to stretching, resulting in a reduced gap between the conductive fibers. This reduction enabled the formation of conductive pathways through contact between silver particles. The pathways were formed more quickly than the disconnection of silver nanoparticles along the fiber axis. Consequently, the total resistance of the conductive fibrous membrane decreased. Within the strain range of 3–23%, the conductive fibrous membrane continued to stretch and lengthen. However, the rate at which conductive pathways of the conductive fibers decreased and became slower than that at which silver nanoparticles disconnect along the fiber axis. As a result, the total resistance of the fibrous membrane increased.

**Figure 8:**
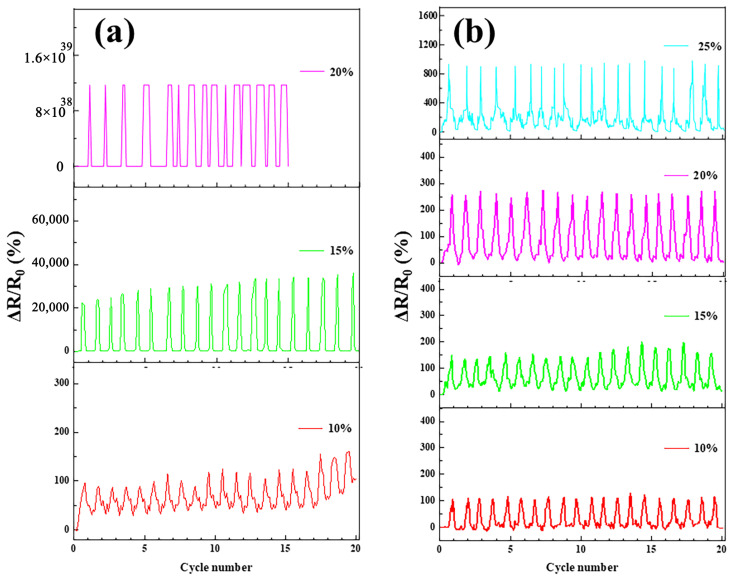
Cyclic strain-sensing properties of (**a**) PVDF and (**b**) AgNPs-PVDF conductive fibrous membranes.

In order to verify the sensing properties of AgNPs-PVDF conductive fibrous membranes with different strain values, the cyclic stretch experiments were conducted. As shown in Figure 8a, Δ*R*/*R*_0_ values increased gradually with the increase in strain values from 10% to 15%. When the strain values reached 20%, the PVDF conductive fibrous membranes were stretch ineffective before a cyclic stretch of 20 times. Moreover, the Δ*R*/*R*_0_ values of PVDF conductive fibrous membranes were not steady, which existed as an obvious fluctuation phenomenon. Compared with PVDF conductive fibrous membranes, AgNPs-PVDF conductive fibrous membranes exhibited steady Δ*R*/*R*_0_ variation in specific cyclic strain processes, as shown in Figure 8b. Moreover, with the increase in strain, Δ*R*/*R*_0_ values of AgNPs-PVDF conductive fibrous membranes increased correspondingly. Based on Figure 4, Figure 6, Figure 7 and Figure 8, it can be found that the AgNPs-PVDF conductive fibrous membrane owned an excellent size and amount of silver particles, high conductivity, and continuous strain-sensing property, which can be used to detect practical strain signals.

**Figure 9:**
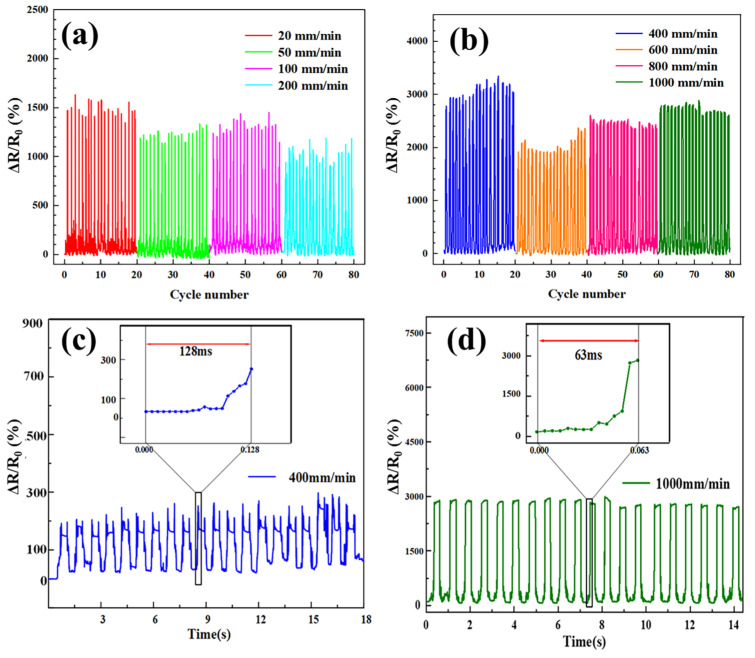
Δ*R*/*R*_0_ variation with various (**a**) low and (**b**) high loading rates, (**c**) response time, and (**d**) Δ*R*/*R*_0_ with 1000 mm/min loading rate for cyclic curves of AgNPs-PVDF conductive fibrous membrane.

Figure 9a,b present the test results of Δ*R*/*R*_0_ following 20 cycles of low-speed and high-speed stretching of AgNPs-PVDF conductive fibrous membranes with the strain of 25% and loading rate ranging from 20 mm/min to 1000 mm/min. It can be observed that Δ*R*/*R*_0_ exhibited minimal fluctuations, steady peak values, and stable signal variation at each stretching speeds. Notably, Figure 9c demonstrated that the AgNPs-PVDF conductive fibrous membrane exhibited the excellent response time of 63 milliseconds at a stretching speed of 1000 mm/min. Figure 9d illustrated the Δ*R*/*R*_0_ performance of the AgNPs-PVDF conductive fibrous membrane with a loading rate of 200 mm/min and 20% strain for 4000 cycles. Notably, the AgNPs-PVDF conductive fibrous membrane demonstrated remarkable durability throughout the test. The Δ*R*/*R*_0_ values increased with the increase in cycle times, which displayed two distinct signals characterized by an “M” shape. This shape was attributed to the mechanical hysteresis of the AgNPs-PVDF conductive fibrous membrane and the reorganization of the silver particles’ conductive network. The loading rate of 200 mm/min realized a balance between excessive speed and sluggishness, allowing the maximum elongation. AgNPs-PVDF conductive fibrous membrane exhibited a certain degree of rebound. With the decrease in Δ*R*/*R*_0_, the stretching machine continued the movement without returning to the original resistance. When the signal reached its peak and the stretching machine returned to its initial position, the signal also returned to its starting point. The prepared AgNPs-PVDF conductive fibrous membrane owned steady sensitivity, linearity, and durability, which built the function base for practical sensing applications.

**Figure 10:**
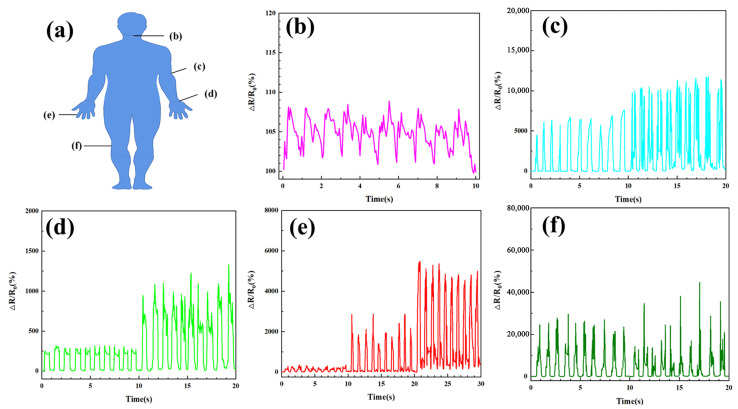
(**a**) A schematic diagram of experimental parts and sensing curves of (**b**) throat, (**c**) elbow, (**d**) wrist, (**e**) finger, and (**f**) knee bending with different angles of AgNPs-PVDF conductive fibrous membrane.

In order to verify the practical sensing properties, AgNPs-PVDF conductive fibrous membranes are placed on throat, elbow, wrist, finger, and knee, to detect corresponding strain signals of the human body with different variation degrees, as shown in Figure 10a. The AgNPs-PVDF conductive fibrous membrane is integrated into the throat with adhesive tape to investigate small-scale human motion. The repeatable Δ*R*/*R*_0_ signals were tested via the sensor when the volunteer spoke the words “Hello”. Obviously, the signals can be easily distinguished steadily during the repeatable process, as shown in Figure 10b. Figure 10c exhibits that the AgNPs-PVDF conductive fibrous membrane was used to detect an elbow bending process from 0° to 45° and 90°. The effective monitoring suggests that the AgNPs-PVDF conductive fibrous membrane strain sensor could be used to identify bending degree information by observing Δ*R*/*R*_0_ signals. Besides the throat and elbow, the motion of the wrist can also be detected. As shown in Figure 10d, Δ*R*/*R*_0_ signals measured the wrist bending process from 0° to 45° and 90°. With the increase in bending degree, Δ*R*/*R*_0_ signals increased correspondingly, indicating the excellent sensing response rate. Fingers are the parts with a high freedom of movement in the human body. Figure 10e shows the AgNPs-PVDF conductive fibrous membrane sensor which is also used to detect the bending degree via mounting on finger. When the finger bent from 0° to 45° and 90°, three diacritical Δ*R*/*R*_0_ curve parts are corresponding to the bending degrees of the finger. To investigate the viability as a strain sensor for detecting high-scale human motion, the strain sensors are integrated into the knee. The strain sensor effectively detected the bending process of the knee.

Figure 10 demonstrates the high sensitivity of the conductive fiber in detecting motion signals, facilitating the accurate detection of diverse movements of finger, wrist, elbow, knee, and throat vibrations. Consequently, the AgNPs-PVDF conductive fibrous membrane exhibits exceptional sensing capabilities. The practical sensing application in Figure 10 proves the excellent sensing properties of AgNPs-PVDF conductive fibrous membranes. Moreover, the preparation of AgNPs-PVDF conductive fibrous membranes on the basis of the preparation of the porous PVDF fibrous membrane and the reduction reaction of silver particles was also verified feasibly and effectively. The investigation of the conductive PVDF fibrous membrane with high conductivity via electrostatic spinning builds and extends the material and functional base for the practical application of functional fibers in the field of man–machine interaction.

## 4. Conclusions

In this investigation, a new kind of conductive polyvinylidene fluoride fibrous membrane with high conductivity was successfully prepared via electrostatic spinning. In order to realize the high absorption amount of Ag^+^ and obtain sufficient silver particles on PVDF fiber surfaces, the porous PVDF fibrous membrane was constructed via the addition and dissolution of PVP into PVDF electrostatic spinning solutions. Based on the reduction reaction process of conductive parts from Ag^+^ to silver particles, the porous PVDF fibrous membrane was changed to an AgNPs-PVDF conductive fibrous membrane. According to microstructure and functional groups’ analysis, a large amount of silver particles was connected tightly with porous PVDF fibers, realizing the formation of a stable conductive network. The AgNPs-PVDF conductive fibrous membrane exhibited low resistance, high conductivity, high sensitivity, and mechanical strength. Additionally, the AgNPs-PVDF conductive fibrous membrane exhibited linear current–voltage characteristics. The cyclic tensile release tests indicated that the conductive fibers possess favorable repeatability, stability, and reproducibility with 4000 cyclic stretches and a strain of 20%. The maximum gauge factor value can reach 24.3 during the strain process from 0% to 23%, which builds a base of excellent electrical sensing properties. Therefore, the prepared AgNPs-PVDF conductive fibrous membrane possessed potential as a wearable electronic device for detecting human movements including movements of the finger, wrist, elbow, knee, and throat vibrations. The excellent conductivity and sensitivity of the AgNPs-PVDF conductive fibrous membrane can be attributed to the stable network structure formed by the incorporation of silver elements, which provide a novel method and unique technical method for the preparation of wearable conductive fibers in the field of man–machine interaction.

## Data Availability

Not applicable.

## References

[1] Li Y., Samad Y., Taha T., Cai G., Fu S., Liao K. (2016). Highly flexible strain sensor from tissue paper for wearable electronics. ACS Sustain. Chem. Eng..

[2] Ge G., Huang W., Shao J., Dong X. (2018). Recent progress of flexible and wearable strain sensors for human-moti on monitoring. J. Semicond..

[3] Liao X., Liao Q., Yan X., Liang Q., Si H., Li M., Wu H., Cao S., Zhang Y. (2015). Flexible and highly sensitive strain sensors fabricated by pencil drawn for wearable monitor. Adv. Funct. Mater..

[4] Zymelka D., Yamashita T., Takamatsu S., Itoh T., Kobayashi T. (2017). Printed strain sensor with temperature compensation and its evaluati on with an example of applicati ons in structural health monitoring. Jpn. J. Appl. Phys..

[5] Li Y., Huang P., Zhu W., Fu S., Hu N., Liao K. (2017). Flexibl e wire-shaped strain sensor from cotton thread for human health and motion detection. Sci. Rep..

[6] He Z., Zhou G., Byun J., Lee S., Um M., Park B., Kim T., Bok S., Chou T. (2019). Highly stretchable multi-walled carbon nanotube/thermoplastic polyurethane composite fibers for ultrasensitive, wearable strain sensors. Nanoscale.

[7] Zhou G., Byun J., Jung Y., Mun B., Jin H., Dong S., Hyun M., Chou S., Wei T. (2017). Highly sensitive wearable textile-based humi dity sensor made of high-strength, singl e-walled carbon nanotube/poly (vinyl alcohol) filaments. ACS Appl. Mater. Interfaces.

[8] Ma Y., Liu N., Li L., Hu X., Zou Z., Wang J., Luo S., Gao Y. (2017). A highly flexible and sensitive piezoresi stive sensor based on MXene with greatly changed interlayer distances. Nat. Commun..

[9] Di J., Zhang X., Yong Z., Li D., Li R., Li Q. (2016). Carbon-nanotube fibers for wearable devices and smart textiles. Adv. Mater..

[10] Yan C., Wang J., Kang W., Cui M., Wang X., Foo C., Chee K., Lee P. (2014). Highly stretchable pi ezor esistive graphene-nanocelulose nanopaper for strain sensors. Adv. Mater..

[11] Luo Y., Li Y., Sharma P., Shou W., Matusik W. (2021). Learning human–environment interactions using conformal tactile textiles. Nat. Electron..

[12] Song Z., Li W., Bao Y., Han F., Gao L., Xu J., Ma Y., Han D., Niu L. (2018). Breathable and skin-mountable strain sensor with tunable stretchability, sensitivity, and linearity via surface strain delocalizati on for versatile skin activities recognition. ACS Appl. Mater. Interfaces.

[13] Amjadi M., Yoon Y., Park I. (2015). Ultra-stretchable and skin-mountable strain sensors using carbon nanotubes-Ecoflex nanocomposites. Nanotechnology.

[14] Sun B., Long Y., Liu S., Huang Y., Ma J., Zhang H., Shen G., Xu S. (2013). Fabrication of curled conducting polymer microfibrous arrays via a novel electrospinning method for stretchable strain sensors. Nanoscale.

[15] Costa P., Carvalho M., Correia V., Viana J., Lanceros-Méndez S. (2018). Polymer nanocomposite-based strain sensors with tailored processability and improved device integration. ACS Appl. Nano Mater..

[16] Fu X., Al-Jumaily A., Ramos M., Meshkinzar A., Huang X. (2019). Stretchable and sensitive sensor based on carbon nanotubes/polymer composite with serpentine shapes via molding technique. J. Biomater. Sci. Polym. Ed..

[17] Gong H., Cai C., Gu H., Jiang Q., Cheng Z. (2021). Flexible and wearable strain sensor based on electrospun carbon sponge/polydimethyl siloxane composite for human motion detection. RSC Adv..

[18] Seyedin S., Zhang P., Naebe M., Si Q., Razal J. (2018). Textile strain sensors: A review of the fabricati on technologies, performance evaluati on and applications. Mater. Horiz..

[19] Tang Z., Jia S., Wang F., Bian C., Chen Y., Wang Y., Li B. (2018). Highly-stretchable core-sheath fibers via wet-spinning for wearable strain sensors. ACS Appl. Mater. Interfaces.

[20] Li W., Zhou Y., Wang Y., Jiang L., Ma J., Chen S., Zhou F. (2020). Core-sheath fiber—Based wearable strain sensor with high stretchability and sensitivity for detecting human motion. Adv. Electron. Mater..

[21] Li W., Zhou Y., Wang Y., Li Y., Jiang L., Ma J., Chen S. (2020). Highly stretchable and sensitive SBS/graphene composite fiber for strain sensors. Macromol. Mater. Eng..

[22] Ryu S., Lee P., Chou J., Rong R. (2015). Extremely elastic wearable carbon nanotube fiber strain sensor for monitoring of human motion. Acs Nano.

[23] Yue X., Jia Y., Wang X., Zhou K., Shen C. (2020). Highly stretchable and durable fber-shaped strain sensor with porous core-sheath structure for human motion monitoring. Compos. Sci. Technol..

[24] Shen L., Yu L., Wang M., Zhu X., Hsiao M., Benjamin S. (2015). Green fabrication of Ag coated polyacrylonitrile nanofibrous composite membrane with high catalytic efficiency. J. Nanosci. Nanotechnol..

[25] Li X., Wang M., Wang C., Cheng C., Wang X. (2014). Facile immobilization of Ag nanocluster on nanofibrous membrane for oil/water separation. ACS Appl. Mater. Interfaces.

